# Abuse and Diversion of Immediate Release Opioid Analgesics as Compared to Extended Release Formulations in the United States

**DOI:** 10.1371/journal.pone.0167499

**Published:** 2016-12-09

**Authors:** Janetta L. Iwanicki, S. Geoff Severtson, Heather McDaniel, Andrew Rosenblum, Chunki Fong, Theodore J. Cicero, Matthew S. Ellis, Steven P. Kurtz, Mance E. Buttram, Richard C. Dart

**Affiliations:** 1 Rocky Mountain Poison and Drug Center, Denver Health and Hospital Authority, Denver, Colorado, United States of America; 2 National Development and Research Institutes, Incorporated, New York, New York, United States of America; 3 Department of Psychiatry, Washington University School of Medicine, St. Louis, Missouri, United States of America; 4 Center for Applied Research on Substance Use and Health Disparities, Nova Southeastern University, Fort Lauderdale, Florida, United States of America; University of Washington, UNITED STATES

## Abstract

**Background:**

Therapeutic use and abuse of prescription opioids in the United States increased substantially between 1990 and 2010. The Centers for Disease Control estimated deaths related to pharmaceutical opioids reached nearly 19,000 in 2014. Of prescription opioids sold, 10% are extended release (ER) and 90% immediate release (IR). However, most regulations and interventions have focused on decreasing ER abuse. Our objective was to compare rates of abuse and diversion of ER and IR opioid analgesics over time using multiple surveillance programs.

**Methods:**

Rates of abuse and diversion of ER and IR opioid formulations were compared using data from four surveillance programs in the Researched Abuse, Diversion and Addiction Related Surveillance (RADARS^®^) System. Data were evaluated from 2009 through 2015, and Poisson regression used to compare IR and ER opioid cases over time.

**Results:**

From 2009 to 2015, IR opioids were prescribed at a rate 12 to 16 times higher than ER. In the Poison Center Program, population-adjusted rates of Intentional Abuse for IR were 4.6 fold higher than ER opioids (p<0.001). In the Drug Diversion Program, population-adjusted rates of diversion were 6.1 fold higher for IR than ER opioids (p<0.001). In the Opioid Treatment Program, population-adjusted rates of endorsements for abuse were 1.6 fold higher for IR opioids than ER (p = 0.002). In the Survey of Key Informants' Patients Program, population-adjusted rates of endorsements for abuse were 1.5 fold higher for IR opioids than ER (p<0.001).

**Conclusions:**

Between 2009 and 2015, IR opioids were prescribed at a much higher rate than ER opioids. Results from four surveillance programs show population-adjusted rates of prescription opioid abuse were markedly higher for IR than ER medications. For the greatest public health benefit, future interventions to decrease prescription opioid abuse should focus on both IR and ER formulations.

## Introduction

Therapeutic use of prescription opioids increased substantially between 1990 and 2010. In concert, abuse and diversion of opioids increased dramatically, resulting in increased contacts with poison centers, visits to emergency departments, admissions to substance abuse treatment centers, and deaths. [1–3] The Centers for Disease Control (CDC) estimated deaths related to pharmaceutical opioids reached nearly 19,000 in 2014. [4] The epidemic of prescription opioid abuse remains an alarming public health concern with severe sequelae and massive public health costs. [5–9]

Several interventions to decrease prescription opioid abuse have focused predominantly on extended release (ER) formulations. The United States Food and Drug Administration (US FDA) expressed strong concerns for the potential of increased serious side effects associated with ER medications, often available in higher milligram concentrations per unit dose. A Risk Evaluation and Mitigation Strategy (REMS) program was developed by the FDA for ER and long-acting (ER/LA) opioids, focusing on risk management via provider education on safe prescribing practices, patient counseling, and patient education. Additionally, the FDA and pharmaceutical companies have focused on developing abuse deterrent formulations (ADFs) to decrease ER opioid abuse. However, the US market share for ER opioids is only 10%, while immediate release (IR) products account for 90% of opioid analgesic prescriptions dispensed. [10,11] Previous research suggests a majority of people who abuse prescription opioids initiated their abuse with IR medications, commonly prescribed for acute pain by primary care physicians. [12–14] Despite their ubiquity, most IR formulations are not subject to REMS or other similar regulations.

Given the wide availability of IR opioids, and their common use to treat acute pain in primary care settings, we hypothesized they contribute greatly to the prescription opioid abuse epidemic. We aimed to compare rates of abuse and diversion of ER to IR opioid analgesic formulations in the US using multiple surveillance programs.

## Materials and Methods

Rates of abuse and diversion of ER and IR opioid formulations were compared using data from the Researched Abuse, Diversion and Addiction Related Surveillance (RADARS^®^) System, a real-time surveillance system that measures prescription drug abuse and diversion for specific products across the US. RADARS System is comprised of a mosaic of programs, each with an independent principal investigator (PI) and unique methodology, which gather data from different populations and provide multiple views on prescription drug abuse. RADARS System is independently owned and operated by Denver Health and Hospital Authority, which operates the public hospital for the city and county of Denver. RADARS System is supported by subscriptions from pharmaceutical companies that use the data in reporting to the FDA. Subscribers had no role in conception, execution, or reporting of this analysis. Each program is approved by the institutional review board of the PI’s institution. Data from four RADARS programs (Poison Center (PC), Drug Diversion (DD), Opioid Treatment (OTP), Survey of Key Informants’ Patients (SKIP)) were used. Further details regarding each program have been published. [15]

For this analysis from 2009 through 2015, products were grouped based on formulation as either IR or ER. Active pharmaceutical ingredients (APIs) of interest were oxycodone, hydrocodone, morphine, hydromorphone, oxymorphone, tramadol, and tapentadol.

The Poison Center (PC) Program studies acute health events from calls from the general population, caregivers, and healthcare providers regarding potentially toxic exposures. Trained specialists at each center collect data using a nationally standardized electronic health record. Data are summarized quarterly. Intentional Abuse exposures were defined by the National Poison Data System definition of an exposure resulting from intentional improper or incorrect use of a substance where the patient was attempting to gain a high, euphoria, or another psychotropic effect. PC cases were defined as the sum of exposure calls mentioning at least one drug within the category. Exposure calls where both an IR and ER opioid were mentioned were counted as a case in each group.

The Drug Diversion (DD) Program provides systematic surveillance data on diversion of drugs. Drug diversion officers submit data quarterly on the number of diversion cases within their jurisdiction. Drug diversion officers represent municipal police departments, multi-jurisdictional drug task forces, county sheriffs’ departments, regulatory agencies, state police agencies, prosecutors’ offices, and departments of health. DD reports are defined as the total number of documented drug diversion cases involving products of interest with investigation that results in a written complaint or report.

The Opioid Treatment Program (OTP) monitors the prevalence of prescription opioid abuse among admissions to federally approved opioid agonist treatment programs. The Survey of Key Informants’ Patients (SKIP) Program collects data from patients entering substance abuse treatment programs (excluding methadone programs). At enrollment, each patient is offered the opportunity to complete a standardized self-administered questionnaire on specific prescription drugs abused “to get high” in the past 30 days. Cases are defined in OTP and SKIP programs as the number of survey respondents who endorse at least one opioid product in the category. After second quarter 2011, tramadol was no longer broken out into IR and ER on the questionnaire.

### Statistical Methods

Rates were calculated by taking the number of cases from a program within a covered 3-digit zip code and dividing by the associated denominator. Three separate denominators were used, population, prescriptions dispensed, and grams dispensed. Population rates used estimates by 3-digit ZIP code obtained from the 2000 and 2010 United States Census. Data are extrapolated for each year quarter subsequent to 2010. Quarterly population rates were calculated by dividing the total number of cases by the sum of the population within 3-digit ZIP code. Prescriptions and grams dispensed estimates were obtained from IMS Health (IMS Government Solutions, Inc. a subsidiary of IMS Health, Inc.) for IR and ER opioids for each quarter and for each 3 digit ZIP code within the US. Prescription and grams dispensed rates were calculated by dividing the total number of cases by the sum of the prescriptions or grams dispensed within 3-digit ZIP codes. Grams dispensed were used for analysis instead of morphine equivalent dose (MED) because the precise value of MED vary by source and across active pharmaceutical ingredients (API). These differences can produce large changes in the data and over or under-estimate abuse rates. Additionally, MED is used to determine analgesic effect by titration, which may or may not correlate with the amount of a given opioid sought to achieve when abusing an opioid. [16]

Poisson regression, log-linear regression for modeling case count data [17], was used with an over/under dispersion parameter to compare IR and ER opioid counts over time. Rates were modeled by including the natural log of the denominator (population, prescriptions or grams dispensed) as an offset term. Polynomial terms for quarter were also included in the models and the highest significant term for either drug group and all lower order terms were kept to assess trends. These models were used to calculate the expected rate for the IR and ER groups in fourth quarter 2015

## Results

**Fig 1:**
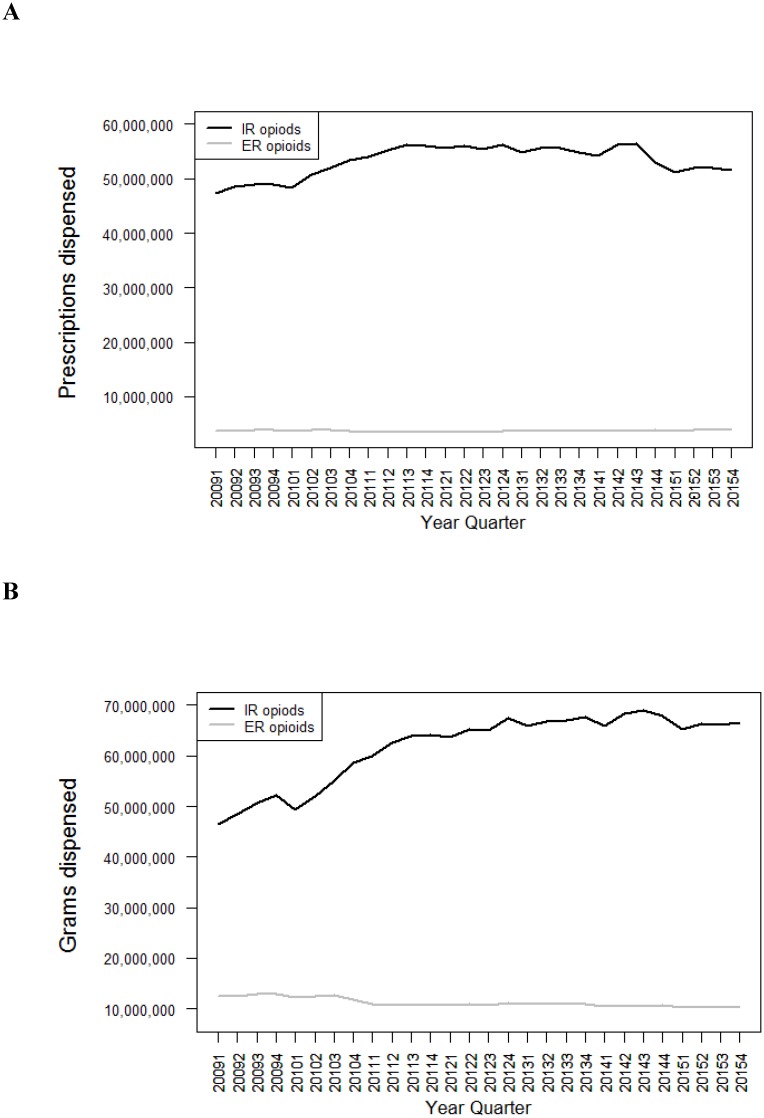
Prescriptions and grams dispensed for immediate release (IR) and extended release (ER) opioid analgesics by quarter and formulation. Data are displayed according to calendar quarter. (A) Number of prescriptions dispensed for IR and ER opioid analgesic formulations. (B) Number of grams of drug dispensed for IR and ER opioid formulations. (IMS Health).

IR opioids were dispensed in much greater quantities than ER opioids, with 12 to 16 times greater prescriptions supplied and 3 to 7 times greater grams dispensed for IR as for ER opioids in each quarter from 2009 to 2015 (Fig 1).

### Poison Center Program Intentional Abuse

**Fig 2:**
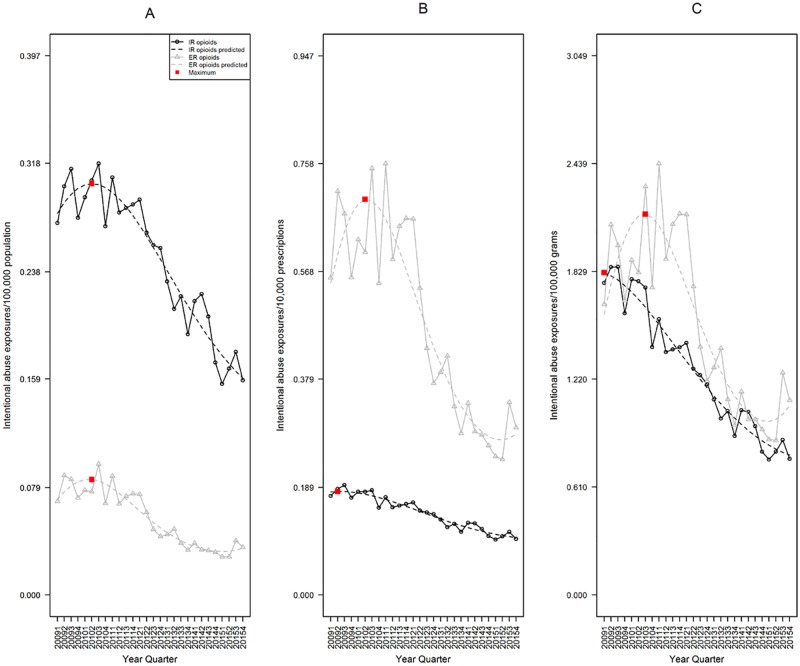
Rates of IR and ER opioid analgesic Intentional Abuse, RADARS System Poison Center Program. Data are displayed according to calendar quarter. (A) Rates of Intentional Abuse adjusted for population; a cubic model was fit for both IR and ER formulations over time. (B) Rates adjusted for prescriptions dispensed; a quadratic model was fit for both IR and ER formulations over time. (C) Rates adjusted for grams dispensed; a quadratic model was fit for both IR and ER formulations over time. The red boxes represent the point with the highest expected rate during the study period.

The population-adjusted rate of Intentional Abuse for IR was significantly higher than ER opioids. In fourth quarter 2015, IR rate was 0.160 exposures per 100,000 population (95%CI 0.145–0.176), while ER was 0.035 (95%CI 0.029–0.042), a 4.6 fold difference (p<0.001, Fig 2A).

The prescription-adjusted rate of ER abuse was significantly higher than IR. In fourth quarter 2015, the IR rate was 0.101 exposures per 10,000 prescriptions (95%CI 0.093–0.110), while ER was 0.283 (95%CI 0.229–0.350), a 2.8 fold difference (p<0.001, Fig 2B).

The grams-adjusted rate of ER abuse was significantly higher than IR. In fourth quarter 2015, the IR rate was 0.790 exposures per 100,000 grams (95%CI 0.715–0.873), while ER was 1.071 (95%CI 0.861–1.332) a 1.4 fold difference (p = 0.013, Fig 2C).

### Drug Diversion

**Fig 3:**
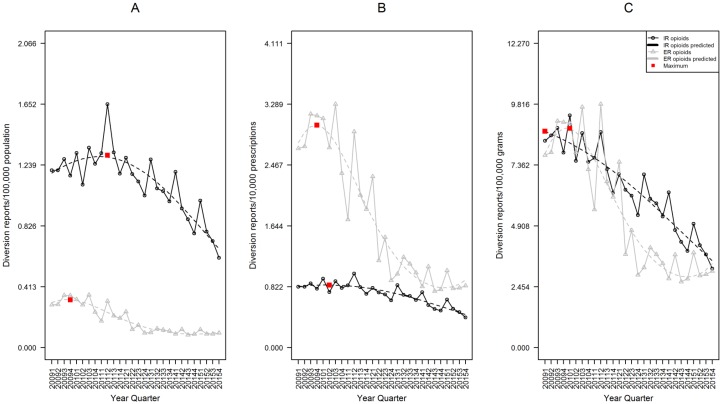
Rates of IR and ER opioid analgesic diversion, RADARS Drug Diversion Program. Data are displayed according to calendar quarter. (A) Rates of endorsement adjusted for population. (B) Rates adjusted for prescriptions dispensed. (C) Rates adjusted for grams dispensed. For each, a cubic model was fit for both IR and ER formulations over time. The red boxes represent the point with the highest expected rate during the study period.

The population-adjusted rate of diversion was significantly higher for IR than ER opioids. In fourth quarter 2015, IR rate was 0.709 diversion reports per 100,000 population (95%CI 0.601–0.836), while ER was 0.116 (95%CI: 0.084, 0.161), a 6.1 fold difference (p<0.001, Fig 3A).

The prescription-adjusted rate of ER opioid diversion was significantly higher than IR. In fourth quarter 2015, the IR rate was 0.446 diversion reports per 10,000 prescriptions (95%CI 0.378–0.525), while ER was 0.916 (95%CI 0.676–1.242), a 2.1 fold difference (p<0.001, Fig 3B).

The grams-adjusted rate of IR opioid diversion was similar to the ER rate, with IR slightly higher than ER. In fourth quarter 2015, the IR rate was 3.501 diversion reports per 100,000 grams (95%CI 2.973–4.123), while ER was 3.386 (95%CI 2.474–4.635), a 1.0 fold difference (p = 0.853, Fig 3C).

### Opioid Treatment Program

**Fig 4:**
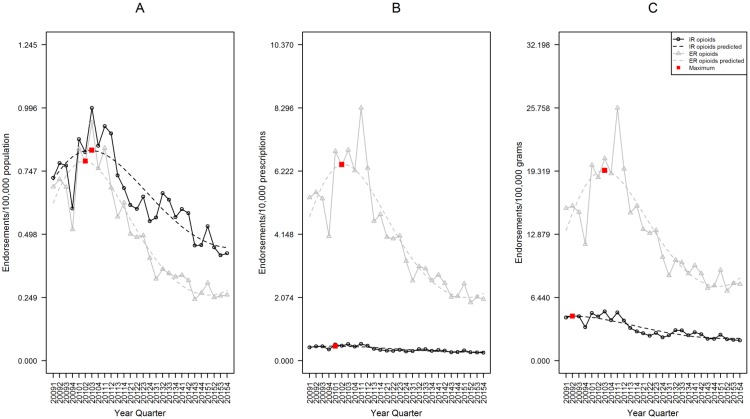
Rates of IR and ER opioid analgesic endorsement, RADARS Opioid Treatment Program. Data are displayed according to calendar quarter. (A) Rates of endorsement adjusted for population. (B) Rates adjusted for prescriptions dispensed. (C) Rates adjusted for grams dispensed. For each, a cubic model was fit for both IR and ER formulations over time. The red boxes represent the point with the highest expected rate during the study period.

The population-adjusted rate of endorsements for abuse was significantly higher for IR opioids than ER. In fourth quarter 2015, the IR rate was 0.446 endorsements per 100,000 population (95%CI 0.369–0.539), while ER was 0.278 (95%CI 0.221–0.349), a 1.6 fold difference (p = 0.002, Fig 4A).

The prescription-adjusted rate of ER opioid endorsements was significantly higher than IR. In fourth quarter 2015, the IR rate was 0.286 endorsements per 10,000 prescriptions (95%CI 0.234–0.349), while ER was 2.207 (95%CI 1.680–2.901), 7.7 fold difference (p<0.001, Fig 4B).

The grams-adjusted rate of ER opioid endorsements was significantly higher than IR. In fourth quarter 2015, the IR rate was 2.295 endorsements per 100,000 grams (95%CI 1.843–2.857), while ER was 8.430 (95%CI 6.415–11.077), 3.7 fold difference (p<0.001, Fig 4C).

### Survey of Key Informants’ Patients

**Fig 5:**
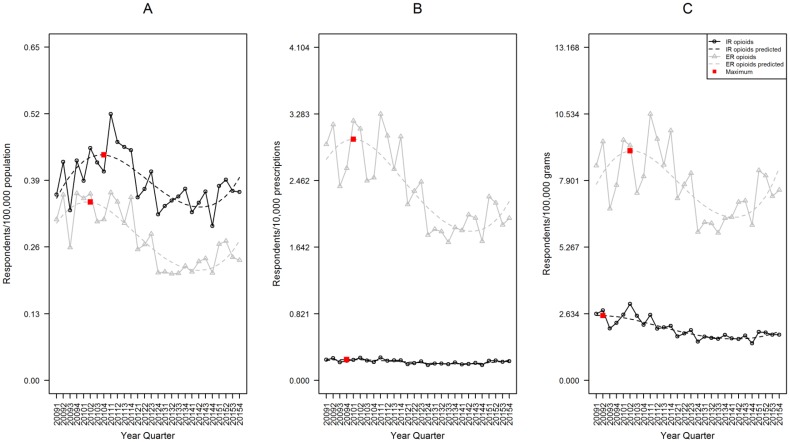
Rates of IR and ER opioid analgesic endorsement, RADARS Survey of Key Informants’ Patients Program. Data are displayed according to calendar quarter. (A) Rates of endorsement adjusted for population. (B) Rates adjusted for prescriptions dispensed. (C) Rates adjusted for grams dispensed. For each, a cubic model was fit for both IR and ER formulations over time. The red boxes represent the point with the highest expected rate during the study period.

The population-adjusted rate of endorsements for abuse was significantly higher for IR opioids than ER. In fourth quarter 2015, the IR rate was 0.396 endorsements per 100,000 population (95%CI 0.352–0.445), while ER was 0.273 (95%CI 0.235–0.317), a 1.5 fold difference (p<0.001, Fig 5A).

The prescription-adjusted rate of ER opioid endorsements was significantly higher than IR. In fourth quarter 2015, the IR rate was 0.247 endorsements per 10,000 prescriptions (95%CI 0.224–0.274), while ER was 2.208 (95%CI 1.889–2.581), a 8.9 fold difference (p<0.001, Fig 5B).

The grams-adjusted rate of ER opioid endorsements was significantly higher than IR. In fourth quarter 2015, the IR rate was 1.941 endorsements per 100,000 grams (95%CI 1.722–2.186), while ER was 8.401 (95%CI 7.126–9.904), a 4.3 fold difference (p <0.001, Fig 5C).

## Discussion

Four different perspectives on prescription opioid abuse and diversion indicate that IR opioids affect a much larger absolute number of individuals than ER opioids. IR opioids are prescribed at a rate 12 to 16 times higher than ER, and dispensed in 3 to 7 times greater milligram quantities, greatly increasing their availability and the number of patients exposed. Despite evaluating unique populations, each program revealed the same trend: abuse and diversion of IR products exceeded ER after adjustment for population. The relative difference between the rate of IR and ER diversion and abuse was greatest in the Drug Diversion and Poison Center Programs, while the smallest difference was seen in treatment programs (OTP and SKIP). The smaller effect in treatment programs suggests that while high-risk experienced people who abuse prescription opioids still abuse IR medications more frequently, they also seek out ER medications at a higher rate than people who abuse opioids more casually.

The high rates of IR abuse have significant public health implications in addressing the prescription opioid epidemic. Our results are consistent with previous work showing the direct relation between increased drug availability and increased abuse. [18,19] Additionally, treatment for acute pain almost always involves an IR opioid analgesic, which may transition to an ER product if chronic treatment is needed. A significant proportion of the population of patients with pain has predisposing factors for addiction, ranging from genetic to psychosocial, regardless of whether they are “appropriately treated” in the clinic. [20] Once patients with risk factors are exposed, some will progress to abuse and addiction, and as the exposed population increases, so does the number of at-risk patients exposed.

While well-intentioned, some federal and state policy makers, as well as payors, have missed a prime opportunity for intervention in the prescription opioid epidemic by focusing primarily on the regulation and control of ER opioids and failing to adequately address IR opioid abuse. [4] This strategy would be expected to address a relatively smaller number of high-risk people who abuse opioids who preferentially seek ER products. However, interventions focusing on IR formulations have the potential to impact a much larger number of individuals, many of whom are initiating opioid abuse. [21] Impeding IR abuse has the potential to halt the natural progression of medication abuse and addiction at a much earlier stage. [22] Therefore, future interventions should target both IR and ER medications. A broader approach will require more resources, but without addressing both formulations, high risk people who abuse opioids who are already addicted are likely to simply switch from ER to IR formulations, and the much larger population abusing IR medications is missed completely. [12,23]

In contrast to population-adjusted rates, prescription-adjusted rates of abuse and diversion were higher for ER than IR opioids across all four programs. However, when adjusted for grams dispensed, the effect size was diminished in the PC and treatment programs (OTP and SKIP), consistent with a high-risk population still preferring to abuse ER medications. This may be due to high milligram doses available in a single tablet, making it easier to obtain large milligram quantities to support their addiction even if they are only abusing orally. [23] Interestingly, when adjusted for grams dispensed, IR opioids were diverted at a higher rate than ER, though this difference was not statistically significant. This suggests that after interventions focusing on ER formulations such as the introduction of ADFs, IR medications may have higher market value on the streets, or potentially IR medications are being diverted in higher milligram dose forms than in the past. [24] Data from the website StreetRx.com, where users enter the price paid for drugs purchased on the street, show that IR oxycodone has a typical street price 30–35% higher per milligram than ER oxycodone as of 2015. [25]

Rates of abuse and diversion peaked in all four programs by the end of 2010, matching trends seen in previous research. [5,26] With the exception of the Survey of Key Informants’ Patients Program, rates of abuse and diversion declined between 2010 and 2015 for both IR and ER formulations in all programs. In most programs, if current trends continue it is possible that prescription-adjusted rates of abuse and diversion for IR opioids will exceed ER in the very near future. Additionally, the amount (grams dispensed) of IR opioids rose dramatically between 2009 and 2012, just as grams dispensed of ER gradually declined. This observation suggests a concerning change in practice toward writing larger prescriptions for IR opioids.

There are several possible explanations for the prominent decrease in prescription-adjusted rates for ER medications. Many interventions, including REMS and ADFs [27,28], focused heavily on ER opioids. Patients and healthcare providers may perceive ER medications as more dangerous, with media coverage often focusing on brand-name ER medications. Widely publicized events such as policy changes leading to closing pill mills in Florida [6,7], release of the first ADF ER opioid medication OxyContin [29], and the CDC report of an HIV outbreak in Indiana associated with intravenous use of ER oxymorphone [30] have predominantly focused on risks associated with ER medications. This may lead to more cautious prescribing and patient use of ER medications. Additionally, some people who abuse opioids have switched from their previous ER drug of choice due to ADF formulations and new restrictions to an IR formulation that is easier to obtain and abuse.

There are several limitations to this study. Poison Center Program data are spontaneously reported, and represent a subset of all possible cases. Additionally, the Opioid Treatment Program and Survey of Key Informants Patients Program rely on self-report and accurate product identification by participants. Finally, the Drug Diversion Program represents only a small portion of all nationwide diversion cases. However, despite these limitations, broad geographic coverage of all of these programs [15], independent data sources and methods for each program, as well as similar trends seen throughout all programs, suggest validity of these findings. Finally, while ER and IR opioids are treated as mutually exclusive categories in this analysis, some people who abuse opioids likely use both categories of opioids. While it is true that people who abuse prescription opioids may abuse IR medications, ER medications, or both, the natural history of prescription opioid abuse suggests that many of these people will move back and forth between these three groups over time. We chose to analyze IR and ER independently because the choice of opioid is multi-factorial and often depends on what medications are available, the costs of those medications, and which medications are available in the most desired formulations. Additionally, some people who abuse prescription opioids likely abuse other drugs as well, such as benzodiazepines and heroin, however this polysubstance abuse is beyond the scope of this study.

## Conclusions

Results from four surveillance programs show abuse and diversion of IR opioids affect a much larger absolute number of individuals than ER opioids. IR opioids are prescribed at a much higher rate than ER, and population-adjusted rates of abuse and diversion of IR opioids are higher than for ER in all four programs. However, prescription-adjusted rates for ER medications are higher than those for IR. Trends over time show declining rates of prescription opioid abuse and diversion for both IR and ER opioids since 2010 in most programs, but prescription-adjusted rates are declining much more rapidly for ER than for IR medications. Future interventions to decrease abuse of prescription opioids in the US should focus on both IR and ER formulations.

## Supporting Information

S1 File Poison Center Program analysis dataset. (CSV)

S2 File Drug Diversion Program analysis dataset. (CSV)

S3 File Opioid Treatment Program analysis dataset. (CSV)

S4 File Survey of Key Informants’ Patients analysis dataset. (CSV)
